# Teaching empathy to medical students

**DOI:** 10.1111/tct.13557

**Published:** 2022-12-22

**Authors:** Gemma McNally, Enam Haque, Sarah Sharp, Harish Thampy

**Affiliations:** ^1^ University of Manchester Medical School Manchester UK

## Abstract

**Background:**

Empathy is a core skill essential to patient‐centred care. Yet a decline in empathy throughout undergraduate medical education is well recognised. The mainstay of existing teaching approaches to foster medical students' empathic ability has largely focused on the cognitive domain of empathy within the context of communication skills learning. However, there is growing evidence for educational initiatives that better evoke students' affective emotional responses.

**Approach:**

A student‐led educational initiative was created to enhance medical students' understanding of empathy through undertaking different experiential approaches. Eight medical students were invited to participate in an empathy workshop that involved two experiential approaches: personal simulation, and patient narrative, selected given their expected focus on the affective domain.

**Evaluation:**

A subsequent focus group discussion explored students' reactions, learning, and intended future change in practice. Discussions were coded and analysed for descriptive themes. Both initiatives were reported to generate students' empathic responses. Personal simulation evoked more affective domains whilst patient narratives additionally created cognitive empathic insight into the impact of the patient's condition. Students also reported intended changes to their future consulting practices.

**Conclusions:**

Although this is a small‐scale exploration of medical students' experiences of empathy‐related teaching initiatives, it offers insight into how each initiative could target different aspects of empathy. Data support the use of even brief experiential teaching to improve medical students' empathy during their undergraduate training. We provide recommendations for clinical educators who are considering designing similar initiatives within their undergraduate medical curricula.

## 1 BACKGROUND

Modern medicine recognises the importance of holistic patient care. Effective communication relies on a patient‐centred approach, to enable shared decision‐making. Empathy is a core skill in patient‐centred consultations in which the clinician must try to ‘enter the patient's world, to see the illness through the patient's eyes’.^1^ Patients who feel their doctors are empathetic divulge more about their symptoms and concerns, increasing diagnostic accuracy.^2^ The literature, however, describes a decline in empathy as students progress through medical school.^3^

Riess defines empathy as a ‘complex capability enabling individuals to understand and feel the emotional states of others, resulting in compassionate behaviour’.^4^ This definition encompasses two components of empathy, commonly recognised in the literature: affective and cognitive. Affective empathy is a personal trait that creates emotional responses through the ability to relate, understand and join another person's experiences. Cognitive empathy, on the other hand, is a skill that students acquire and develop through training and may involve ‘detached concern’ in which understanding another's experience need not evoke an emotional response.^5^

The cognitive domain of empathy has conventionally been seen as a target for intervention, as it is considered a skill and therefore amenable to modification.^6^ This has typically been delivered through communication skills training using simulated patients with learners adopting language such as ‘I am sorry to hear that’ to convey empathy.^7^ However, overreliance on such stock phrases and structures can create mechanistic consultations with inauthentic displays of empathy.

Questions, therefore, remain about how best to inculcate empathy among medical students. A systematic review of 18 papers identified several interventions that were evaluated for effectiveness in developing students' empathy.^6^ The majority utilised validated empathy questionnaires to explore the effect of the chosen intervention. Other initiatives have included patient narratives, writing compositions, arts‐based approaches, communication skills training, patient interviews and personal simulation. Patient narratives and personal simulations particularly enhanced learners' affective empathy. The latter approach arises from the theory that humans create meaning and learn from their experiences and interactions.^8^ There has been no direct comparison of different experiential approaches. Furthermore, existing work has largely focused on demonstrating quantitative impacts as measured through empathy scales, with a recent review calling for further qualitative work in this area.^9^ Less is known about how different approaches are accepted or perceived by learners, particularly concerning the affective domain. We therefore designed an educational innovation aiming to address this gap by exploring medical students' experiences and perspectives of undertaking two different affective‐focused empathy interventions.

We … designed an educational innovation … exploring medical students' experiences and perspectives of undertaking two different affective‐focused empathy interventions.

## 2 APPROACH

This was a single‐site educational initiative among third‐year medical students conducted as a student‐led project.  Students were invited to participate in a voluntary 2‐h workshop focusing on schizophrenia, with the opportunity to experience two different types of initiatives, personal simulation and patient narratives. These were selected given their anticipated focus on the affective domain.

Students were invited to participate in a voluntary 2‐h workshop focusing on schizophrenia.

Eight students (four male and four female) consented to participate via email and re‐consented at the start of the workshop. They were split randomly into two groups of four; half of the group initially participated in a personal simulation session and the other half in the patient narrative session. They then swapped sessions.

The personal simulation session required participants to listen individually to a recorded auditory hallucination for 15 min. The recording used intrusive, negative thoughts that were created by a mental health professional for authenticity. This approach has previously been shown to enhance learners' empathy.^10^ The patient narrative session, on the other hand, asked participants to listen to a patient's account of their journey through the diagnosis of their condition. Students were then asked to reflect on this experience through small group discussion. Table 1 further describes the two initiatives.

**TABLE 1:** Description of the two initiatives

	Simulation	Patient narrative
Content:	Intrusive thoughts. Extracts from the audio: ‘Look at you, stupid, stupid’ ‘Jump, jump now! … go on, do it do it.’ ‘pointless, worthless’ ‘I can see you’ ‘she knows, she knows’ The audio had voices overlapping one another and included hissing and other incomprehensible sounds.	Story from an IT professional with a diagnosis of schizophrenia. Extracts from the narrative: The job was gone, the gun was loaded, and a voice was saying, ‘You're a waste, give up now, do it now’. It was a command, not a suggestion, and what mattered at that moment was not where the voice was coming from, but how assured it was, how persuasive. ‘All I remember then is a knock on the bedroom door and my wife, she sits down on the bed and hugs me, and I'm holding the gun in my left hand, down here, out of sight’. ‘When I think of all that happened, I just can't believe she's still with me, you have to understand, for so many years I was hearing her say terrible, nasty things that she wasn't saying’. ‘I was so broken’ and ‘I just thought, “Well, I'm a weirdo, I'll never be normal.”’
Length	15 min (audio was 03:38 put on a loop) Students were told they could stop the audio early if they felt too distressed.	5 min to read the narrative and 15 min of reflective questioning led by the facilitator

Our institution's ethics tool confirmed that this study did not need formal ethics approval though attention was paid throughout to ethical matters. Participation was voluntary and informed consent was obtained for both workshop participation and the use of focus group data within publications, per the Declaration of Helsinki.

## 3 EVALUATION

All eight participants attended a 1‐h focus group discussion to explore students' experiences and perspectives of each experience, moderated by GM.

Focus group discussions were recorded, transcribed and then thematically analysed by the lead author through in‐depth coding to derive descriptive themes.^11^ We identified three overarching themes. (1) Experiential learning evokes students' empathic responses; (2) experiential interventions may promote future change in students' practices; (3) current empathy teaching is insufficient and should shift to experiential learning opportunities. These are described in Table 2, with exemplar quotations provided for each.

**TABLE 2:** Themes and Corresponding Data

Theme	Description	Quotes
Experiential learning evokes students' empathic responses	In both interventions, students valued the immediacy of direct engagement in the patient perspective and reported that the richness of each deepened their understanding and insight into the patient's experiences.	‘You feel it a lot more rather than when you're reading about someone else's experiences’. ‘You can immerse yourself in the hallucination’.
	Whilst simulation triggered the affective empathy domain in which students reflected on the impact of a condition using a personal lens, the narrative seemed to additionally trigger cognitive aspects of empathy, encouraging students to challenge pre‐held conceptions of what was likely to be important for the patient	The audio (simulation) was more distressing, but then the patient narrative kind of showed how much of a wide impact it had on their lives. ‘The story (patient narrative) made you realise that this could be completely different to how it would impact someone else's life’. ‘[The patient] said that he doesn't think it's an important question that his childhood was bad, which I would've thought would have been the most important’. ‘I will no longer think that my guess is right with what would help the patient’.
Experiential interventions may promote future change in students' practices	Students reported that both sessions served as powerful drivers for changing their consultation approaches to become more patient centred. Students reported a deeper understanding of the importance empathy plays in patient interactions. Students also reflected upon intentions to alter their language and communication style when consulting with patients in the future.	‘[Asking] what it is like for them on a daily basis’ ‘Normally they just say they have hallucinations, and you'd just write it down as a symptom, but you might not explore it in the same way’. ‘I learned how to adapt my word choice a bit more’. ‘I think doing this just with one condition would encourage students to put themselves in people's shoes for other conditions as well’.
Current empathy teaching is insufficient and should shift to experiential learning opportunities.	Participants described perceived deficiencies with current communication skills training, voicing concerns that their current teaching did not prepare them for different situations. Respondents unanimously felt that medical school teaching should use experiential sessions to deepen students' appreciation of empathy and for them to authentically display this in interactions with patients.	‘We're taught to say certain phrases which are the same for each condition’. ‘A lot of people will just say a phrase like ‘sorry to hear that’ just to tick the box rather than there actually being any sort of empathy’. ‘It would help you learn the theory a lot more… it will stick in your mind and would help link the theory and experience together, as opposed to just a lecture’. ‘When people are saying “sorry to hear that” there will [now] be more of a meaning behind it’.

## 4 IMPLICATIONS

This innovative educational session explored medical students' experiences of two different empathy‐focused teaching interventions. Whilst both generated students' empathic responses, personal simulation appeared to evoke more affective domains whilst patient narratives additionally created cognitive empathic insight into the patient's lived experience. Our students also commented that this workshop expanded their understanding beyond that of the specific clinical condition covered in the session suggesting the transferability of learning across clinical contexts. Though we acknowledge that students' reported change in future practice was self‐anticipated based on their immediate reflections, others have reported that even brief targeted interventions can have lasting effects on students' ability to display empathy.^12^

Whilst both generated students' empathic responses, personal simulation appeared to evoke more affective domains whilst patient narratives additionally created cognitive empathic insight into the patient's lived experience.

Our school is currently making revisions to our Year 1 and Year 2 stage of the programme with a planned increase in experiential learning opportunities. In light of this evaluation, we are now incorporating community‐based IPE empathy focussed teaching utilising lessons learnt from this work supplemented by in‐practice early clinical practice. This evaluation has also identified onward questions for future larger‐scale research to identify the best ways to integrate empathy teaching into undergraduate medical curricula. Although we recruited students from the start of their clinical phase, based on findings that empathic decline is most pronounced at this point,^3^ the optimal time to introduce this topic remains unclear. Furthermore, further work is needed to compare one‐off and longitudinal approaches, to help inform future curriculum development. Lastly, the question of how best to balance and integrate classroom‐based empathy teaching with real encounters in the clinical environment requires further exploration.

Although we recognise the small‐scale exploratory nature of this work, the results still provide a useful foundation from which to make onward recommendations for clinical educators considering delivering similar interventions. These are summarised in Table 3. Similar innovations could be easily implemented across institutions in different regions of the world and cultural contexts with minimal resource implications. Finally we believe that this approach offers transferability to other health professions education programmes as well as fostering inter‐professional learning.

**TABLE 3:** Recommendations for designing empathy‐focused teaching sessions

Focus interventions around real patient scenarios that offer students a window into a patient's lived experiences
Combine different empathic interventions within teaching episodes to trigger students' affective and cognitive empathy domains
Create a safe learning environment to allow students to safely share and challenge preconceptions
Incrementally introduce more challenging scenarios integrated across the curriculum to help learners apply empathy development from the classroom to real clinical settings
Offer post‐session facilitated reflective debrief to manage learners' heightened emotions triggered by the experience
Co‐create initiatives between students and staff to inform effective educational design and delivery

This approach offers transferability to other health professions education programmes.

## CONFLICT OF INTEREST

The authors have no conflict of interest to disclose.

## ETHICS STATEMENT

Standards of the Declaration of Helsinki were maintained; all data were collected anonymously, and consent for the workshop was gained through email and then again at the start of the workshop to participate in the workshop. Written consent was then obtained again for publication of the data in a journal.
